# Can Patients with Parkinson’s Disease Use Dry Powder Inhalers during Off Periods?

**DOI:** 10.1371/journal.pone.0132714

**Published:** 2015-07-14

**Authors:** M. Luinstra, A. W. F. Rutgers, H. Dijkstra, F. Grasmeijer, P. Hagedoorn, J. M. J. Vogelzang, H. W. Frijlink, A. H. de Boer

**Affiliations:** 1 Department of Clinical Pharmacy, Martini Hospital, Groningen, The Netherlands; 2 Department of Pharmaceutical Technology and Biopharmacy, University of Groningen, Groningen, The Netherlands; 3 Department of Neurology and Clinical Neurophysiology, Martini Hospital, Groningen, The Netherlands; University of Toronto, CANADA

## Abstract

Because of its rapid onset of action, pulmonary administration of levodopa is an interesting alternative to oral administration for the rescue treatment of Parkinson’s disease patients in an off period. We studied the ability of Parkinson’s disease patients to operate a dry powder inhaler (DPI) correctly during an off period. We used an instrumented test inhaler with three different resistances to air flow to record flow curves and computed various inhalation parameters. We observed that all (13) patients were able to generate pressure drops > 2 kPa over the highest resistance and 10 out of 13 patients achieved at least 4 kPa. Inhaled volumes (all resistances) varied from 1.2 L to 3.5 L. Total inhalation time and the time to peak inspiratory flow rate both decreased with decreasing inhaler resistance. Twelve out of thirteen patients could hold their breath for at least five seconds after inhalation and nine could extend this time to ten seconds. The data from this study indicate that patients with Parkinson’s disease will indeed be able to use a dry powder inhaler during an off period and they provide an adequate starting point for the development of a levodopa powder inhaler to treat this particular patient group.

## Introduction

Parkinson’s disease is a degenerative disorder of the central nervous system, causing various movement related and psychiatric symptoms. Levodopa and dopamine agonists are effective in alleviating the motor symptoms of the disease, but a high variability in levodopa absorption from the gastrointestinal tract after oral administration causes fluctuations in the plasma concentration of the drug [1]. In more advanced Parkinson’s patients, this often results in fluctuations between ‘on periods’, in which the Parkinson’s disease symptoms are well controlled, and ‘off periods’, in which the Parkinson’s disease symptoms are poorly controlled [2]. Off periods are characterised by an extensive variety of complaints, such as decreased mobility, bradykinesia, tremor, autonomic symptoms, sensory symptoms and psychiatric disorders [3]. A delayed onset of effect of levodopa after oral administration due to irregular gastrointestinal absorption can cause a delayed or even failing return of the motor function after an off period [4], which is a significant burden to patients.

Therefore, an increased, faster and more reproducible absorption for levodopa as that provided by the currently used oral medication is desired. Pulmonary administration may offer an attractive alternative to oral administration, due to its immediate presentation of the drug to the absorption membrane, the large size of the absorption membrane and relatively low metabolic activity in the lungs [5]. The pulmonary epithelium can be targeted with inhaled drug aerosols, for instance, from a dry powder inhaler (DPI) [6]. Previous studies in rats [7] have already shown a rapid onset of action after pulmonary administration of levodopa. This makes inhalation of the drug particularly interesting for the rescue treatment of Parkinson’s disease patients in an off period. From the storage stability point of view, a dry powder inhalation product is preferable over a nebulised formulation as levodopa in solution has a poor stability [8]. However, correct operation of a passive DPI requires that the patient will be able to perform an appropriate inhalation manoeuvre, not only for adequate aerosol generation by the DPI, but also for good deposition of the aerosol in the target area. Therefore, the inspiratory flow manoeuvre performed by the patient is crucial for achieving the desired improvement in bioavailability.

Hardly anything is known about Parkinson’s disease patients’ abilities to operate a DPI. Especially in off periods, bradykinesia and rigidity might diminish the inspiratory muscle strength and, thereby, reduce the ability to use a DPI appropriately [9]. This expectation is strengthened by the finding of Guedes et al [10] that the mean maximal inspiratory pressure (MIP) of Parkinson’s disease patients in an off period is lower than the MIP of healthy volunteers. Although the exact implications of this finding regarding the ability to operate DPIs are unclear, it does question the feasibility of rescue treatment by dry powder inhalation for Parkinson’s patients in an off period.

For that reason, the aim of our study was to assess the ability of Parkinson’s disease patients to use a DPI suitable for the administration of a high dose of levodopa during an off period. For this study, a test inhaler with three different resistances to air flow around the resistance of the RUG’s Cyclops disposable DPI [11] was used. We monitored how the test inhaler was handled and we recorded the inspiratory flow rates generated by 13 Parkinson’s disease patients while they were in an off period. Flow rate and breath hold recordings were evaluated by comparing them with the requirements for adequate operation of the Cyclops as well as achieving efficient aerosol deposition.

## Materials and Methods

### Sample size

This study is a non-therapeutic observational study designed to obtain information on the ability of Parkinson’s patients to handle and operate a dry powder inhaler correctly during off periods. Since we only wanted to obtain insight into the ability of Parkinson’s patients to use the inhaler correctly during off periods, no sample size calculations were performed. A sample size of 15 patients was considered to be sufficient.

### Study design

**Fig 1:**
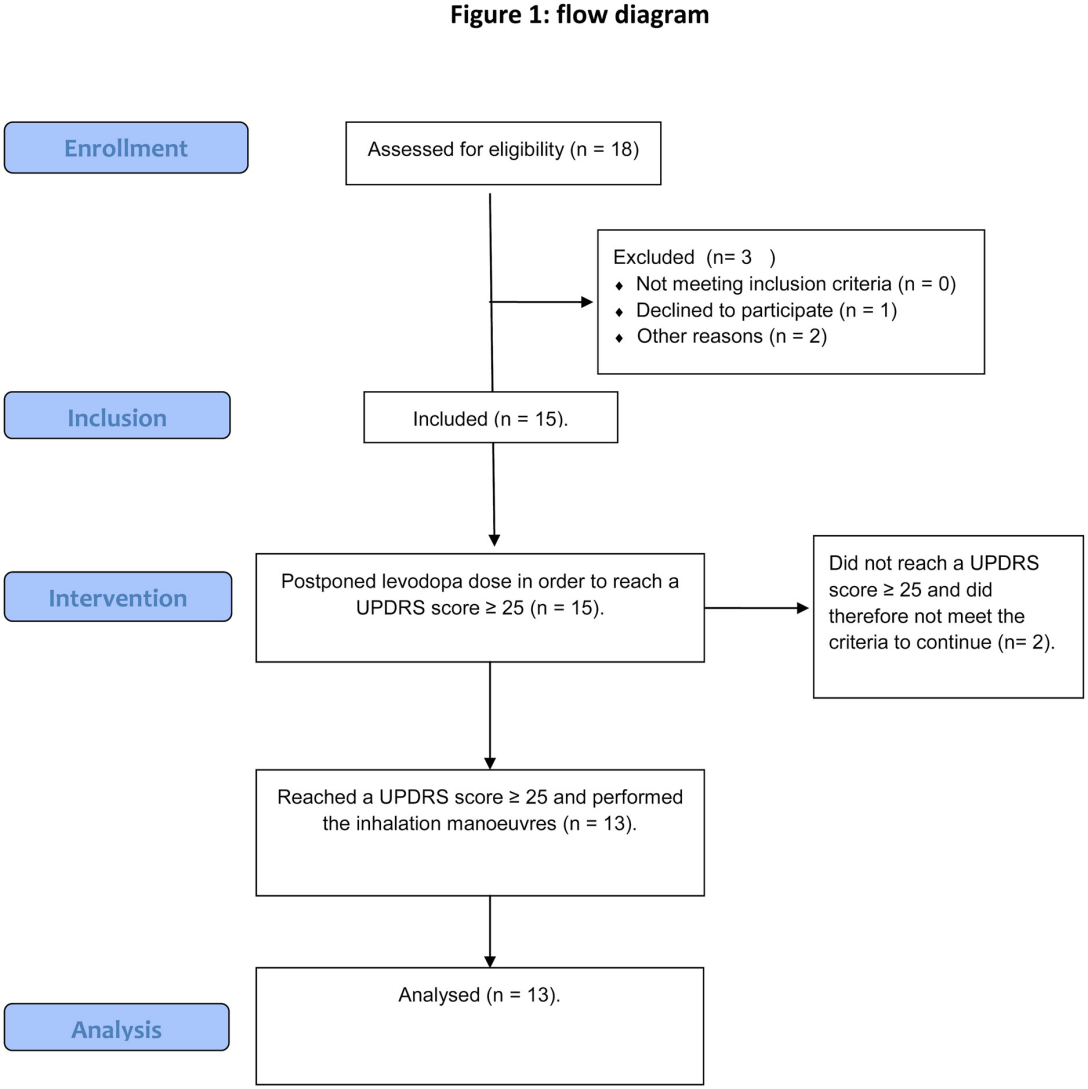
Flow diagram study layout.

The study was registered at the Dutch Trial Register (Nederlands trial register; number NTR4202). At baseline each potential participant was examined to determine eligibility for participation in this study. This included a written informed consent, information on their co-morbidities (pulmonary disease) and collection of their Parkinson’s disease history. A flow diagram of the study design is shown in Fig 1.

### Patient recruitment

Patients were recruited from the Martini hospital in Groningen (the Netherlands). A hospital neurologist with Parkinson expertise selected suitable patients according to the defined inclusion criteria. The participants received patient information and an informed consent form. Patients were allowed to contact an independent physician for advice. The patient, neurologist and researcher signed the informed consent form when consent was given.

Only patients that met all pre-set criteria were included in the study. Patients eligible to participate were diagnosed with Parkinson’s disease according to the UK Parkinson’s disease society Brain Bank Clinical Diagnostic Criteria, at least 2 years of levodopa use, predictable off periods, recognisable off periods for themselves and others and at least 18 years old.

Patients with active pulmonary disease or with cognitive dysfunction, which precludes good understanding of instructions and/or informed consent, were excluded. The study was performed in the Martini Hospital Groningen, the Netherlands.

### Study protocol

**Fig 2:**
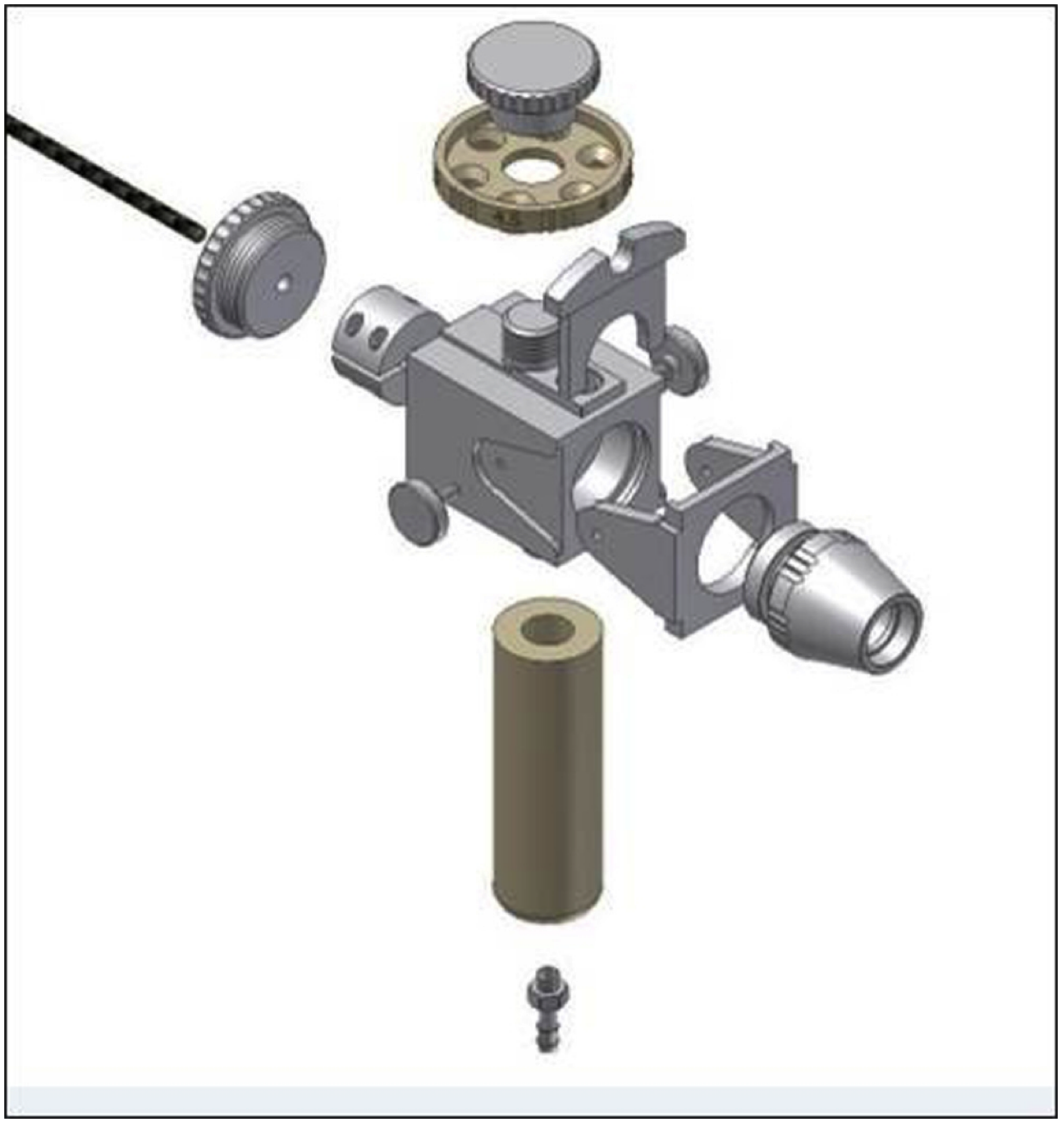
Test inhaler with the rotating disk having the three resistance orifices on top.

The study was conducted with an in-house designed and manufactured instrumented test inhaler (Fig 2) with three exchangeable air flow resistances, R1, R2 and R3. The resistance to air flow was controlled with a rotatable ring, which consisted of three orifices with different diameters placed closely against the inlet channel. This resulted in resistances to air flow of 0.061 kPa^0.5^.min.L_N_^-1^ (R1); 0.048 kPa^0.5^.min.L_N_^-1^ (R2); 0.037 kPa^0.5^.min.L_N_^-1^ (R3) respectively. These resistances are considered medium/high to high, according to the ERS/ISAM task force consensus statement [12].

The test inhaler was equipped with a differential pressure gauge (PD1 with MC2A measuring converter; Hottinger, Baldwin Messtechnik, Darmstadt, Germany) which measured the pressure drop generated by the patient across the test inhaler during inhalation. Previously, flow rate versus pressure drop relationships for all three resistances were recorded using the same differential pressure gauge and a thermal mass flow meter (Model 5863S, Brooks instruments B.V., Ede, The Netherlands). During the inhalation experiments the pressure gauge was linked to a computer equipped with LabView software (National Instruments BV, The Netherlands), which was used to calculate and store the inspiratory flow rate as function of the inhalation time using the previously recorded flow rate versus pressure drop relationships for each of the three resistances.

Patients were scheduled around a planned levodopa gift and were asked to postpone the planned levodopa dose until they felt off. During the waiting time, the test procedure was explained, in which the patient received instructions on how to use the test inhaler. The patients were instructed to sit straight, hold the inhaler in the correct position and exhale completely prior to inhalation, which was scored subjectively as moderately, sufficient or good exhalation, based on the observation of the researcher. Good was defined as complete exhalation, sufficient was defined as almost complete exhalation and moderately was defined as a short exhalation.

After bringing the inhaler to the mouth, the patients were instructed to inhale as firmly and as long as possible, in order to study their maximum attainable pressure drop over the different resistances of the test inhaler. Thereafter, they were asked to hold their breath for 10 seconds before exhalation. These instructions were given verbally. The opportunity to do a few test inhalations with the test inhaler previous to the experiments was offered to all patients. During the inhalation the patient was allowed to see the flow curve on the computer screen. This provided the opportunity to illustrate and explain how the inhalation manoeuvre could be improved.

As soon as the patient felt off, a Unified Parkinson’s disease rating scale section III motor examination (UPDRS III motor score) was performed by the Parkinson neurologist to assess the severity of the off period. The UPDRS is a tool to quantify the severity of Parkinson’s disease based on the rating of multiple symptoms [13] and is the primary outcome measure in most clinical trials of Parkinson’s disease therapeutics [14]. The UPDRS consists of six sections, in which all items (unless otherwise indicated) are rated from zero (normal) to four (severely affected). The UPDRS III motor examination section is a 14-item rating of motor signs. It rates tremor, facial and generalized bradykinesia, and performance on several straightforward tasks [15]. Since motor symptoms (rigidity, bradykinesia) are major symptoms during an off period, the UPDRS III motor examination section is useful to assess the clinical state of motor function immediately before the inhalation test is started.

As soon as the neurologist assured that the patient was off (UPDRS III motor score ≥ 30), recording of the inhalation manoeuvres was started. If the UPDRS III motor score was < 30, the patient had to wait for 2 hours at maximum. When the UPRDS III motor score reached a value ≥ 25 after this 2 hour waiting period, the recording of the inhalation manoeuvres was started. Patients with UPDRS III motor score < 25 after two hours waiting were not included in the study. All patients performed at least two, but no more than three inhalations per air flow resistance. All patients performed the inhalation manoeuvres in the same order starting with R1 (highest), followed by R2 and finally R3 (lowest). Relevant inspiratory parameters from these flow curves were computed and averaged per patient per resistance. After finishing the test inhalations, the patient took his/her oral levodopa dose.

### Ethics

The study was approved by an official Dutch ethics committee (Regionale Toetsingscommissie Patientgebonden Onderzoek). All participants provided written informed consent for their participation in this study.

### Data analysis

An ANOVA one way statistical test was used to compare the mean inhalation characteristics between the different resistances (R1 versus R2, R1 versus R3 and R2 versus R3) of the test inhaler. We corrected for multiple comparison by using the Tukey’s multiple comparison technique. The adjusted p-values are considered significant when they are <0.05. Statistical analyses were performed with GraphPad, Prism version 6.0e.

## Results

### Subjects

Fifteen Parkinson’s patients were included in this study. Two patients did not reach a UPDRS III motor score ≥ 25 and were, therefore, not included in the study. The flow curves of the other 13 patients were accepted for analysis. Of these 13 patients, 12 (92%) were able to perform an inhalation manoeuvre according to the instructions. One patient was not able to exhale completely prior to inhalation. This study was performed between April 2014 and October 2014. Age, height, weight and UPDRS III motor score prior to the inhalation manoeuvre are shown in Table 1.

**Table 1:** Characteristics of Parkinson’s patients with a UPDRS III score ≥ 25 prior to inhalation.

Age (y)	68.0 (55.0–80.0)
Height (m)	1.82 (1.65–1.93)
Weight (kg)	78.0 (60.0–95.0)
Time since diagnosis Parkinson’s disease (yr)	13 (3–22)
UPDRS score prior to inhalation	34 (25–53)

(N = 13; 6 male; 7 female). Median (min–max) are shown.

### Inhalation manoeuvre

It was observed that, for most Parkinson’s patients, it is difficult to perform an inhalation manoeuvre correctly according to the instructions the first time. The entire manoeuvre consisted of a couple of successive actions, and it was particularly difficult for the patients to perform these actions in the right order. After practicing, sufficient improvement was obtained by all patients but one, who showed the tendency to exhale through the inhaler, even after repeated exercise. This patient performed the inhalation manoeuvre correctly only when the instructor kept giving instructions till the moment of exhalation.

Before the actual inhalation, all patients were asked to exhale completely to residual volume. Of the 13 patients, 10 patients performed a good exhalation manoeuvre, 2 patients exhaled sufficiently and 1 patient exhaled only moderately according to previously given definitions.

**Fig 3:**
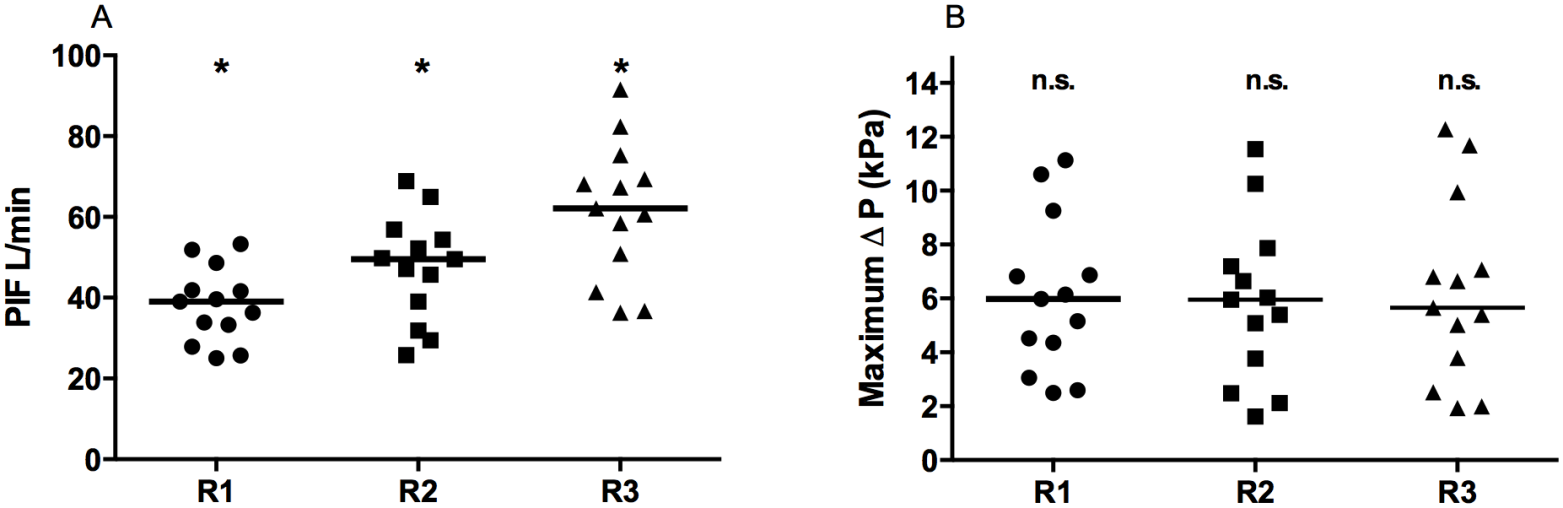
PIF and maximum Δ P for the three resistances to air flow. (A) PIF; (B) maximum Δ P. Each data point represents the mean of a single patient. The line represents the median value of all data points per resistance. Statistical difference between R1 and R2, R2 and R3 and R1 and R3 was tested with the one-way ANOVA (p<0.05). * indicates statistically significant results, n.s. indicates no significant different results.

Fig 3A and 3B present the overall mean values of the participants for the peak inspiratory flow rate (PIF) and the peak pressure drop (Δ P) reached by all patients for each the three resistances to air flow. There was no significant effect of the resistance on the peak pressure drop. The variation for all three resistances is considerable, ranging from a minimum of approximately 2 kPa to a maximum of approximately 12.5 kPa.

The PIF and its variation both increased with decreasing resistance to air flow. The effect of the resistance on the PIF is statistically significant. Across all patients, the minimum PIF ranged from 25.1 to 25.8 and 36.3 L/min, whereas the maximum PIF ranged from 53.3 to 68.9 and 91.5 L/min for R1, R2 and R3, respectively.

**Fig 4:**
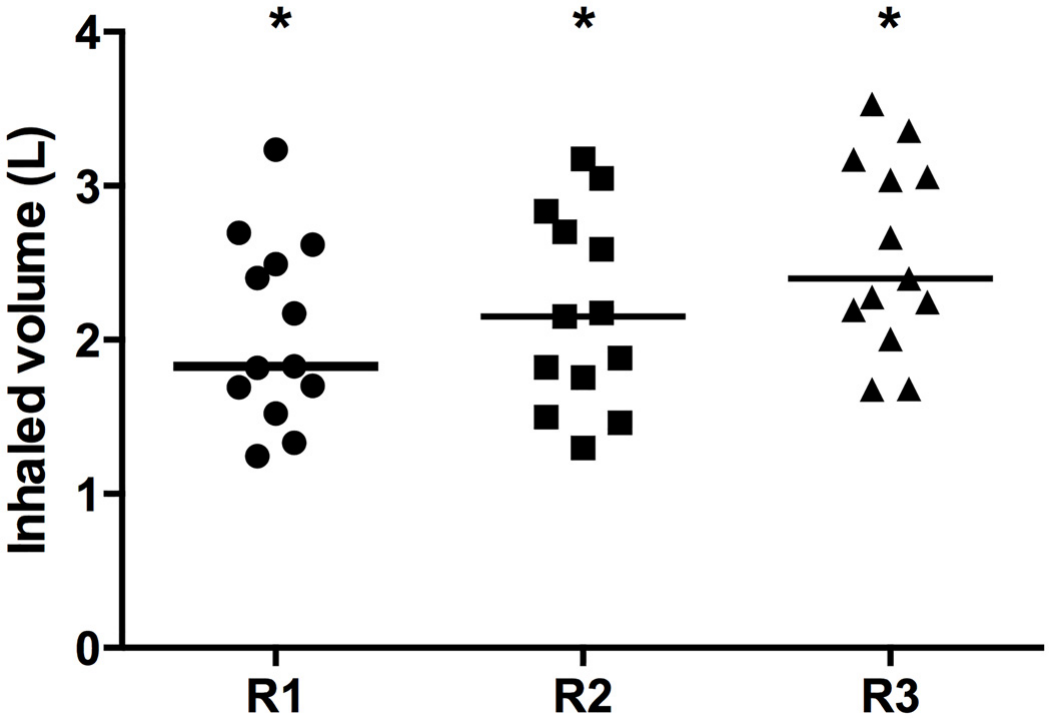
Inhaled volume for the three resistances to air flow. Each data point represents the mean of a single patient. The line indicates the median value of all data points per resistance. Statistical difference between R1 and R2, R2 and R3 and R1 and R3 was tested with the one-way ANOVA (p<0.05). * indicates statistically significant results.

For all three resistances to air flow there is a large inter-patient variation of inhaled volumes (Fig 4). Inhaled volumes ranged from a minimum of 1.2 L for R1 to a maximum of 3.5 L for R3.

**Fig 5:**
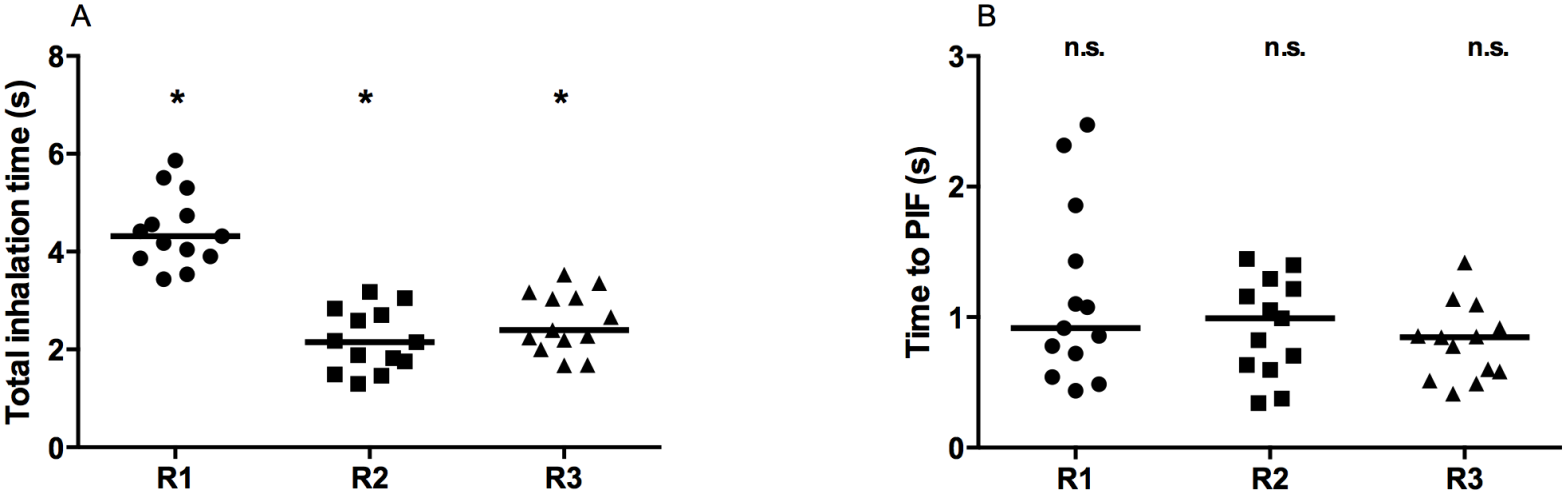
Total inhalation time and time to PIF for the three resistances to air flow. (A) total inhalation time, (B) time to PIF. Each data point represents the mean value of a single patient. The line indicates the median value of all data points per resistance. Statistical difference between R1 and R2, R2 and R3 and R1 and R3 was tested with the one-way ANOVA (p<0.05). * indicates statistically significant results, n.s. indicates no significant different results.

Both the total inhalation time (Fig 5A) and the time to PIF (Fig 5B) were found to decrease with decreasing resistance to air flow. The individual total inhalation time varied from a minimum of 2.8 s for R3 to a maximum of 5.9 s for R1.

For both the total inhalation time and the time to PIF, the ranges are broad due to the high variability between patients. For R1, for two patients, a maximum time to PIF > 2 seconds was observed.

### Breath hold time

Of all 13 patients, 12 were able to hold their breath for at least 5 seconds after inhalation and 9 of them were able to extend the breath hold time to 10 seconds. One patient was not able to hold his breath at all.

### Other observations

All patients tried to sit up as straight as possible with their rigidity. Some patients had antecollis or thoracal kyphosis, which caused a curved upper body.

After instruction, all patients except one were able to hold the test inhaler in horizontal position. A present tremor was not problematic for holding the test inhaler in the right position. Although some patients initially tended to exhale through the inhaler during the test session, training could prevent this, except for one patient. No adverse events or other safety issues were reported during the study. Most patients mentioned that it took longer than in an ‘at home situation’ to become off, probably due to the provoked stress of the unusual test situation.

## Discussion

For successful systemic administration of levodopa peripheral lung deposition is desired. The large surface area available for absorption, a limited metabolic activity and the leaky nature of the membrane, warrant a rapid and high absorption of the drug in the systemic circulation [16][17].

Satisfactory drug deposition in the peripheral region of the lungs from a DPI can only be attained when several conditions are met. It all starts with correct handling of the DPI. For patients with Parkinson’s disease having an impaired motor function, preparing the DPI for inhalation has to be intuitive, require a minimal number of steps and the whole operation should be simple to perform. Next, during inhalation of the dose, the flow rate through the inhaler and the inhaled volume of air have to be sufficiently high to entrain the powder formulation from the dose system and disperse it into an aerosol with the appropriate aerodynamic size distribution. However, from a lung deposition point of view, high flow rates must be avoided as this results in substantial deposition in the oropharynx by inertial impaction [18]. Mouth-throat deposition reduces the dose available for lung deposition. A proper balance between what is needed for adequate aerosol delivery from the inhaler and drug deposition in the lungs is, therefore, mandatory. Reaching the peripheral airways with the aerosol by convective transport furthermore requires the inhalation of a sufficiently high volume of air. Finally, the residence time for the particles must be long enough for settling by sedimentation and to make contact with the airway wall. Particles residing insufficiently long have a great chance of being exhaled again [18]. Therefore, after inhalation, a certain breath hold period is needed [6].

Also, the properties of the inhaled levodopa aerosol must meet certain requirements to reach the peripheral airways. These requirements include a high mass fraction of the dose in the appropriate aerodynamic particle size. From previous studies, e.g. Usmani et al. [19] it can be concluded that the range of aerodynamic particle diameters suitable for effective deep lung deposition at relatively low flow rates is limited to 1–3 μm. Particles larger than 3 μm inhaled at flow rates > 30 L/min are primarily deposited in the oropharynx. Usmani et al. showed that oropharyngeal deposition of 3 μm particles inhaled at 67 L/min is of the same order of magnitude as that of 6 μm particles inhaled at 31 L/min. Aerosol particles should neither be too small, as their settling velocity decreases exponentially with their diameter. Particles ≤ 1 μm have a considerable chance of being exhaled again, even after a breath hold of 10 s [18].

Having defined the conditions for efficient deep lung deposition regarding particle size and flow rate, the specifications for the inhaler can be set. Such an inhaler has to deliver a sufficiently high mass fraction of the dose as an aerosol in the required size range of 1–3 μm at a flow rate of approximately 30–50 L/min. Furthermore, to reach the most distal airways, the bolus of the aerosol has to be released into the first volume fraction of inhaled air. Although the total volume to be inhaled equals preferably the vital capacity for maximal peripheral deposition, it practically depends on what patients with Parkinson’s disease can attain and the inhaler performance has to be adjusted to that. Previously, the Cyclops^®^ disposable high dose DPI as a member of the Twincer^®^ family was described [11]. The basic design of this inhaler, developed for pulmonary administration of aminoglycosides, will also be used for the administration of levodopa to patients with Parkinson’s disease. In brief, the inhaler consists of three plate like-parts and an aluminium blister with the drug. Access to a dose is obtained by simply pulling the lidding foil off the blister which makes the inhaler ready for inhalation in one single step. It is anticipated that this one step operation to activate a dose will not cause insuperable difficulties for Parkinson’s patients, which are mostly elderly patients. The Cyclops has a high resistance to air flow of 0.060 kPa^0.5^.min.L_N_^-1^ which limits the inhaled flow rate to only 34 L/min at 4 kPa [11]. Hence, the Cyclops can meet the prerequisites for effective deep lung deposition of levodopa when dispersion of this drug is good at 4 kPa, or lower, and the bulk of the dose is released within the first volume fraction of air inhaled. For significant peripheral delivery, the dose should be discharged from the inhaler within the first 1 to 1.5 L of inhaled air [20] and an additional volume of 300 mL (chase air) is required to transport the aerosol into the peripheral airways [21]. A total inhaled volume of approximately 1.3–1.8 L may therefore be sufficient.

Knowing from the *in vitro* deposition experiments what is needed for good dispersion enables us to evaluate the recorded flow curves of the patients with Parkinson’s disease in good perspective. In this study, we observed that all patients were able to generate a pressure drop of 2 kPa, or more, when the resistance was the same as that of the Cyclops. Only 3 out of 13 Parkinson’s patients could not attain a pressure drop ≥ 4 kPa over the test inhaler. Their maximal values were 2.5, 2.6 and 3.1 kPa respectively which is still sufficiently high for the Cyclops. Six patients created pressure drops > 6 kPa when the resistance was the same as that of the Cyclops, and the maximal value achieved was 11.1 kPa. Previous studies with tobramycin have shown that powder dispersion in the Cyclops is already highly efficient at 2 kPa [11]. In a next study we will show that good powder dispersion with the Cyclops at the same pressure drop, corresponding with a low flow rate of only 25 L/min, can also be obtained for levodopa. A higher pressure drop than needed for good fine particle dose delivery from the Cyclops is unwanted since this causes an increase in the oropharyngeal deposition, although even at 11 kPa (the maximal value obtained for R2) the corresponding flow rate through the Cyclops is still only 56 L/min.

We also observed that all patients inhaled a volume of air > 1.3 L through R2 and this may be sufficient for significant peripheral deposition [20] [21]. For the development of a levodopa powder and inhaler combination, the volume needed to emit the entire dose from the inhaler should be adapted to this volume. Since it is known that a higher volume will ensure better drug penetration into the distal airways [22], patients must be trained to exhale before the inhalation as to maximise the inhaled volume.

Regarding the breath hold time, our study showed that for all three resistances to air flow, most (12/13) patients were able to hold their breath for 5 second and 9 of the patients were able to extend the breath hold time to even 10 seconds. The achieved breath hold time of 5 seconds is theoretically sufficient for significant sedimentation of particles of 2 and 3 μm in the peripheral airways [17]. For efficient sedimentation of 1 μm particles, a breath hold period of at least 10 seconds is required. Thus, during the development of a levodopa DPI, a primary particle size of 2–3 μm in the aerosol should preferably be aimed for.

Based on the inhalation parameters we observed in our study, the use of a DPI adapted to the inhalation capacity during an off period seems a feasible option for most Parkinson’s patients.

In addition to the inhalation performance, several other factors have to be taken into account when evaluating whether dry powder inhalation can be a good alternative to oral administration of levodopa to Parkinson’s patients. Although our patients gave the impression that they must be capable of performing a simple action like pulling the cover foil from a blister in the Cyclops in an off period, we have not actually tested this yet. All our patients but one were also capable of holding the test inhaler in the correct horizontal position but they were under continuous surveillance during the tests and were told to do better when they performed incorrectly. Most of our patients also mentioned that it took longer than normally to become off and they may have been under stress to perform well during the tests. Surveillance and stress may be absent in the home situation however. An important aspect of consideration is the cognition of Parkinson’s patients. A few patients in our study encountered problems in performing the different steps of the entire inhalation manoeuvre in the correct order. Particularly exhaling through the device prior to inhalation was observed and although training improved the situation considerably, it must be expected that without permanent surveillance during their inhaler use at home, patients may fall back to incorrect use again.

## Conclusions

Based on the inhalation manoeuvres we observed in our study, the use of a DPI adapted to the inhalation capacity during an off period seems a suitable option for most Parkinson’s patients. One patient was not able to perform a correct inhalation manoeuvre. For this patient, inhalation of levodopa during an off period may not be appropriate. The inhalation data gathered in this study can be used for the successful development of a levodopa DPI for the rescue treatment of this particular patient group.

## Supporting Information

S1 File Trend statement checklist. (PDF)

S2 File Data sheet Parkinson’s inhalation characteristics. (XLS)

S3 File Research protocol ‘ applicability of Parkinson’s patients to use a DPI’. (PDF)
