# Supplementary material for: Can Patients with Parkinson’s Disease Use Dry Powder Inhalers during Off Periods?
Source: PLoS One. 2015 Jul 14;10(7):e0132714. doi: 10.1371/journal.pone.0132714 (PMC4501550; doi:10.1371/journal.pone.0132714)
Supplement: S3 File — (PDF) [file pone.0132714.s003.pdf]

Research protocol

Investigation of the applicability of dry powder inhalation in patients with Parkinson's disease.

PROTOCOL TITLE ‘Investigation of the applicability of dry powder inhalation in patients with Parkinson’s disease’.

**Protocol ID**	**NL45210.099.13**
**Title in Dutch**	**Onderzoek naar de toepasbaarheid van droog poeder inhalatie bij patiënten met de ziekte van Parkinson.**
**Short title**	**Dry powder inhalation in patients with Parkinson’s disease.**
**Version**	**2**
**Date**	**26-11-2013**
**Coordinating investigator/project leader**	**Prof. dr. H.W. Frijlink, Department of Pharmaceutical Technology and Biopharmacy, University of Groningen. 050-363 3285, h.w.frijlink@rug.nl**
**Principal investigators (in Dutch: hoofdonderzoeker)**	**Dr. A.W.F. Rutgers, MD, Neurologist. Martini Ziekenhuis, Groningen, 050-5247947. A.W.F.Rutgers@mzh.nl**<br>**M. Luinstra, MSc, PharmD. Hospital Pharmacist i.t.**<br>**Martini Ziekenhuis, Groningen, 050-5246722, m.luinstra@mzh.nl**
**Sponsor (in Dutch: verrichter / opdrachtgever)**	**Department of Pharmaceutical Technology and Biopharmacy, Faculty of Mathematics and Natural Sciences, University of Groningen**
**Independent physician(s)**	**Dr. H. Kramer, MD PhD, Pulmonary Physician. Martini Ziekenhuis Groningen, 050-5245930. Henk.Kramer@mzh.nl**
**Laboratory sites**	**Department of Pharmaceutical Technology and Biopharmacy, University of Groningen.**

PROTOCOL SIGNATURE SHEET

Name	Signature	Date
Department of Pharmaceutical Technology and Biopharmacy, Faculty of Mathematics and Natural Sciences, University of Groningen. Head of department.<br>*Prof. dr. H.W. Frijlink*		
Clinical Pharmacy, Martini Ziekenhuis Groningen. Head of department.<br>*Drs. T. Visser, PharmD, Hospital Pharmacist*		
Principal investigator/Physician:<br>*A.W.F. Rutgers, neurologist, MD*		
Principal investigator<br>*M. Luinstra, MSc, PharmD, Hospital pharmacist in training*		
Independent physician(s)<br>*Dr. H. Kramer, MD PhD, Pulmonary Physician.*		

TABLE OF CONTENTS

1. INTRODUCTION AND RATIONALE 8
1.1 Parkinson's disease 8
1.2 Pulmonary administration of levodopa in Parkinson's disease 9
1.3 The Twincer™ DPI 10
1.4 Inhalation therapy in Parkinson's patients 11
1.5 Test inhaler 11
1.6 Motor function examination 12
2. OBJECTIVES 14
3. STUDY DESIGN 15
4. STUDY POPULATION 16
4.1 Population (base) 16
4.2 Inclusion criteria (must meet all inclusion criteria) 16
4.3 Exclusion criteria (excluded if they meet one or more of the following criteria) 16
4.4 Sample size calculation 16
5. METHODS 17
5.1 Study parameters 17
5.1.1 Main study parameters 17
5.2 Test inhaler 18
5.3 Study procedures 18
5.4 Withdrawal of individual subjects 20
5.5 Replacement of individual subjects after withdrawal 20
5.6 Premature termination of the study 20
6. SAFETY REPORTING 21
6.1 Section 10 WMO event 21
6.2 Adverse and serious adverse events 21
6.3 Follow-up of adverse events 22
7. STATISTICAL ANALYSIS 22
7.1 Descriptive statistics 22
8. ETHICAL CONSIDERATIONS 22
8.1 Regulation statement 22
8.2 Recruitment and consent 22
8.3 Benefits and risks assessment, group relatedness 23
8.4 Insurance 23
9. ADMINISTRATIVE ASPECTS AND PUBLICATION 25
9.1 Handling and storage of data and documents 25
9.2 Amendments 25
9.3 End of study report 25
9.4 Public disclosure and publication policy 25
10. STRUCTURED RISK ANALYSIS 26
10.1 Potential issues of concern 26
10.1.1 Previous use of the test inhaler 26

10.1.2 Analysis of potential effect. ... 26
10.1.3 Study population ... 27
10.1.4 Can effects be managed? ... 27
10.2 Synthesis ... 27
11. REFERENCES ... 28
APPENDIX 1 ... 29
The relationships between air flow resistance, flow rate, aerosol generation and lung deposition ... 29
APPENDIX 2 UPDRS (section III) motor examination ... 30

LIST OF ABBREVIATIONS AND RELEVANT DEFINITIONS

ABR	ABR form, General Assessment and Registration form, is the application form that is required for submission to the accredited Ethics Committee (In Dutch, ABR = Algemene Beoordeling en Registratie)
AE	Adverse Event
AR	Adverse Reaction
CA	Competent Authority
CCMO	Central Committee on Research Involving Human Subjects; in Dutch: Centrale Commissie Mensgebonden Onderzoek
CV	Curriculum Vitae
DPI	Dry Powder Inhaler
DSMB	Data Safety Monitoring Board
EU	European Union
EudraCT	European drug regulatory affairs Clinical Trials
FIR	Flow Increase Rate
IC	Informed Consent
IMP	Investigational Medicinal Product
IMPD	Investigational Medicinal Product Dossier
METC	Medical research ethics committee (MREC); in Dutch: medisch ethische toetsing commissie (METC)
MIFR	Mean Inspiratory Flow Rate
PIFR	Peak Inspiratory Flow Rate
(S)AE	(Serious) Adverse Event
Sponsor	The sponsor is the party that commissions the organisation or performance of the research, for example a pharmaceutical company, academic hospital, scientific organisation or investigator. A party that provides funding for a study but does not commission it is not regarded as the sponsor, but referred to as a subsidising party.
SUSAR	Suspected Unexpected Serious Adverse Reaction
UPDRS	Unified Parkinsons Disease Rating Scale
Wbp	Personal Data Protection Act (in Dutch: Wet Bescherming Persoonsgegevens)
WMO	Medical Research Involving Human Subjects Act (in Dutch: Wet Medisch-wetenschappelijk Onderzoek met Mensen

SUMMARY

Rationale: because of limited treatment options with a very rapid onset for Parkinson's disease patients in the *off* period, there is a need for the development of rapid onset options to administrate levodopa, like a pulmonary formulation of levodopa.
The development of a dry powder inhaler (DPI) that suits the needs of Parkinson's patients, based on their inspiratory capacities, is essential. In this study, we will investigate the applicability of dry powder inhalation in Parkinson's patients in order to enable us to develop a DPI specifically for this patient group.
Objective: to determine if a Parkinson's patient can operate a test inhaler correctly during *off* periods, by testing if they can generate a sufficiently large airflow and volume through the test inhaler *theoretically* necessary to disperse a medicinal powder and transport the aerosol into the lower airways during an *off* period.
Study design: non-therapeutic observational study.
Study population: 15 Parkinson's patients.
Main study parameters: the main study parameter is the ability of a Parkinson's patient to use the inhaler correctly during *off* periods. The parameter that describes whether or not a Parkinson's patient is capable of using the inhaler correctly is the inspiratory flow curve (s)he generates trough the inhaler upon inhalation, from which various inspiratory parameters relevant to dispersion and deposition can be calculated. For good performance of the current Twincer™, a single use dry powder inhaler in development for levodopa, a pressure drop between 2 and 4 kPa and a total inhalation time of 1 to 1.5 s are optimal, but ,depending on the performance of the patients in this study further optimisation of the inhaler design is possible.
Nature and extent of the burden and risks associated with participation, benefit and group relatedness: The inhaler to be used is a specially designed dummy without drug or excipient, so the Parkinson's patient will not inhale anything but air during the test. The burden is minimal as the procedures are limited to the performance of a couple of inhalations (maximum 10). Per Parkinson's patient, the test is limited to one test moment that lasts maximally 30 minutes per test moment. This observational study has no specific benefits for the participating Parkinson's patients. Only when performed in this population, information on the inspiratory capacities of Parkinson's patients can be obtained. Since all patients included are familiar with frequent *off* episodes, no new discomfort compared to other *off* episodes is expected. The timeframe between the morning levodopa dose and the postponed levodopa dose is expected to be shorter than the levodopa free period during the night. The patients are used to a levodopa free period and the effect of this levodopa free period on their motor function.

1. INTRODUCTION AND RATIONALE

1.1 Parkinson's disease

About 40.000 people in the Netherlands suffer from Parkinson's disease, which is a progressive neurodegenerative disorder with a lack of dopamine production due to the loss of dopamine producing cells in the substantia nigra. This lack of dopamine causes disruption of motor circuits in the brain resulting in motor function impairments like tremor, rigidity and bradykinesia. At first, symptoms are mild, but these symptoms will progress over time. Treatment with dopaminergic drugs (especially levodopa) will improve motor function but after some years many patients develop motor fluctuations. Motor fluctuation means fluctuation between *off* times (decreased mobility) and *on* times, when symptoms are controlled (often with hyperkinesia). It is estimated that about 40 percent of patients with Parkinson's disease will experience motor fluctuations within 4-6 years after onset of Parkinson's disease (Nutt et al 2008), which negatively impact on quality of life despite optimized oral therapy. Early morning akinesia, in which patients awaken with recurrent symptoms of Parkinsonism after a medication free period during the night, often is an initial motor complication. These patients may notice a delayed symptomatic effect from their oral morning dose of levodopa.
Another common problem in Parkinson's patients is an end of dose detoriation in mobility (*wearing off*): the effect of a given dose of medication does not last until the effect of the next dose. *Wearing off* episodes may also occur during sleep and can cause patients to awaken because of tremor or rigidity, bradykinesia en difficulty turning in bed. During these episodes, an additional dose of levodopa may be tried, but because of the slow rise of plasma levels, it will take some time to return to sleep, i.e. until the medication has effect.
Due to variability in levodopa absorption following oral administration of levodopa, return of the motor function may be delayed (Doi et al, 2012). *Off* times are not limited to decreased mobility, patients are also suffering from sensory symptoms (motor restlessness, pain etc), autonomic symptoms (urinary incontinence) and psychiatric disorders (psychosis, anxiety, depressions).

A current strategy to rescue patients from an *off* episode is the administration of levodopa/benserazide disper per os or apomorphine per subcutaneous injections. However, these options both still have a delayed onset of effect, and administration of subcutaneous apomorphine requires (self) injection. Chen et al (2005) performed a systematic review of intermittent subcutaneous apomorphine injections for the rescue management of motor fluctuations. Their review showed that the onset of effect of

subcutaneously injected apomorphine occurred within 20 minutes, but a more rapid onset is preferable. A major disadvantage of apomorphine is nausea and vomiting. Apomorphine is an effective emetic, which makes it necessary for patients to use anti-emetics on a regular base (Ul Haq et al, 2007).

Delayed gastric emptying seems to be a causative factor for delayed-on in Parkinson's disease (Doi et al, 2012). Also, gastric emptying time is delayed in some Parkinson's disease, especially in patients with motor fluctuations. Dispersible levodopa (levodopa/benserazide) has a shorter Tmax compared with standard tablets (Nyholm D, 2006; Contin, M 1999), because gastric emptying is less dependent on pylorus contraction compared to conventional solid dosage forms (Goole et al, 2009). Therefore it is often used to rescue patients from an *off* episode. But for dispersible levodopa to be effective, the levodopa still needs to be absorbed by the small intestine. Most *off* symptoms improve about 30 – 60 minutes after administration of dispersable levodopa, but in some patients improvement of symptoms will be delayed or there will be no response at all (Jankovic et al 2007). Because of the lack of treatment options with a very rapid onset for Parkinson's disease patients in the *off* period, there is a need for the development of rapid onset options to administrate levodopa, like a pulmonary formulation of levodopa, used as rescue therapy. Rescue therapy is used on an acute, as-needed basis to return patients to an *on* state when they are experiencing an *off* state. Rescue therapy aims at a rapid return to an *on* state in patients with wearing *off* or patients with early morning akinesia.

1.2 Pulmonary administration of levodopa in Parkinson's disease

Bartus et al (2004) showed that an inhalable formulation of levodopa had superior pharmacokinetic properties in a rat model of Parkinson's disease. After pulmonary delivery of levodopa, plasma levels were elevated faster than after oral administration. The study also showed a 3-5 fold dose advantage compared to oral administration. In the *off* period, a Parkinson's patient needs a rapid onset of levodopa effect to provide a rapid relief. Pulmonary administration of levodopa therefore could be beneficial if used as rescue therapy in Parkinson's patients during an *off* period.

The department of Pharmaceutical Technology and Biopharmacy of the University of Groningen has developed the Twincer™ dry powder inhaler (DPI). It is a disposable, ready-to-use device that can deliver high doses of drugs into the lungs, which is a prerequisite for levodopa, which is dosed in milligrams.

1.3 The Twincer™ DPI

The Twincer™ DPI is designed to deliver high doses of drugs. It has been developed by the department of Pharmaceutical Technology and Biopharmacy of the University of Groningen. The Twincer™ has an efficient dispersion principle, based on patented air classifier technology, which can generate high dosages of the medication. This reduces the number of inhalations to one or maximally two with a total administration time of less than a minute including preparation of the inhaler. It also enables a reduction of the influence of the patient's breathing behaviour on drug delivery. The Twincer™ is ready-to-use, the only action the patient (or his/her caregiver) has to undertake is pulling the foil strip from the blister. The Twincer™ also has a conveniently small size of about a credit card. Finally, the Twincer™ is a disposable device. In figure 1, a prototype of the Twincer™ is shown (de Boer et al., 2006).

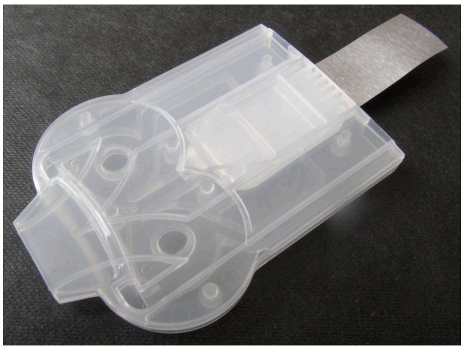

Figure 1: Injection moulded prototype of the Twincer™ DPI

However, the Twincer™, like nearly all currently marketed dry powder inhalers, is a passive device. This means that the energy for the emptying of the dose compartment (entrainment of the powder) and dispersion of the powder into the correct aerodynamic particle size distribution is derived from the inhaled air stream. To deliver sufficient energy for that, it requires that the air flow rate through the inhaler is sufficiently high. Additionally, the inhaled volume has to be sufficiently large for convective transport of the aerosol into the peripheral airways and a certain breath hold period after inhalation (preferably 5 s) is needed to give 1-3 µm particles sufficient time for sedimentation in the smallest airways. Dry powder doses with the Twincer™ are preferably (but not necessarily) administered in one single inhalation manoeuvre. To decide whether the Twincer™ is suitable for Parkinson's patients in the *off* period, it has to be investigated whether these patients can comply with the requirements for good inhalation through this device. If not, adjustment of the dispersion efficacy and emission time for the fine particle dose as well as the air flow resistance of the inhaler device may be needed.

1.4 Inhalation therapy in Parkinson's patients

Little is known about Parkinson's patients abilities and capacities to operate dry powder inhalers (DPIs), especially in *off* periods, when the mobility of these patients is decreased. Canning et al (1997) evaluated exercise capacity, respiratory function and gait in patients with mild to moderate Parkinson's disease. Respiratory function tests showed that the mean maximal inspiratory pressure (MIP) and mean maximal expiratory pressure (MEP) values were not significantly different from the predicted age and gender matched values. Guedes et al (2012) investigated the MIP and MEP in patients with Parkinson's disease during *on* and *off* periods compared to healthy volunteers. Their study showed that men and women with Parkinson's disease had lower MIP and MEP values during the *off* period compared to controls.
Seccombe et al (2011) recruited 19 patients with mild-moderate Parkinson's disease without respiratory disease and assed ventilatory control. They found that MIP values were below the lower limit of normal in 13 out of 19 patients and MEP values were below the lower limit of normal in 15 out of 19 patients.

The studies mentioned above show that there probably are differences in MIP and MEP values in Parkinson's patients compared to healthy controls. To be able to distinguish if and what alterations to the inhaler device should be made to make it more suitable for use in Parkinson's patients during *off* periods, the applicability of dry powder inhalation with the Twincer™ in Parkinson's patients during *off* periods needs to be tested.

1.5 Test inhaler

Figure 2 shows the test inhaler, which has a disk with six different orifices that have varying diameters. By rotation of the disk, each of the different orifices can be used as air inlet to the inhaler. They represent different resistances to air flow: the smaller the orifice diameter, the higher is the air flow resistance. The orifices have been chosen to cover the range of air flow resistances of commercially available dry powder inhalers. In this study, three resistances will be used (one resistance comparable with the resistance of the Twincer^{TM}, one higher and one lower resistance). In Appendix 1, a more extensive explanation is enclosed of the relationships between air flow resistance, flow rate, aerosol generation and lung deposition.

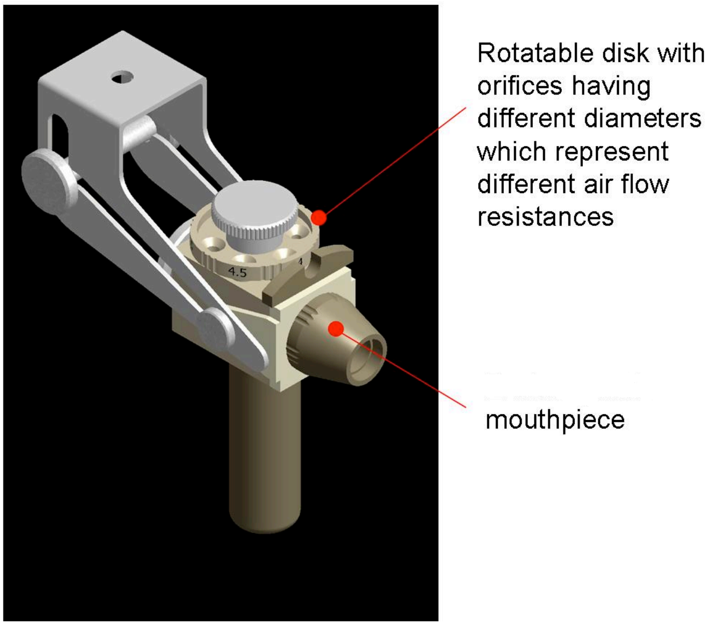

Figure 2: Test inhaler with exchangeable air flow resistance and mouthpiece

In figure 2, the standard conically shaped mouthpiece that will be used is shown. The generated pressure drop across the inlet orifice is measured with a differential pressure gauge (measuring against ambient pressure) and the connection of the inhaler to this pressure gauge is with a flexible air tube through the handle of the inhaler (not shown).

The data obtained with the test inhaler could be used to optimise the Twincer™ DPI for Parkinson's patients. Specific characteristics of the Twincer™ could be adjusted to what Parkinson's patients can do with a DPI, in order to realise a DPI with levodopa that Parkinson's patients can operate during the *off* phase. Such characteristics are for example the resistance, the dose and the emission profile. With this new knowledge we aim to develop a Twincer™ version with levodopa for Parkinson's patients.

1.6 Motor function examination

The Unified Parkinson's Disease Rating Scale (UPDRS) (see appendix 2) is a tool to assess severity of symptoms in Parkinson's disease (Pal et al, 2013). The UPDRS is the primary outcome measure in most clinical trials of Parkinson's disease therapeutics (Tavares et al, 2005). The UPDRS consists of six sections, in which all items (unless otherwise indicated) are rated from zero (normal) to four (severely affected).

Since motor symptoms (rigidity, bradykinesia) are major symptoms during an *off* period, the UPDRS III motor examination section will be useful to assess the clinical state of motor function immediately before the inhalation tests will start. The UPDRS motor examination section is a 14-item rating of motor signs. It rates tremor, facial and

generalized bradykinesia, and performance on several straightforward tasks (Factor et al, 2002). Each item is rated from zero (normal) to four (severely affected).

In this study, an accredited UPDRS scorer will rate the UPDRS III motor section for each patient, immediately before the inhalation tests will start. This allows the researchers to specify the motor function during inhalation. We prefer to start the inhalation tests when the UPDRS III motor score is at least 30 (out of 56). However, if the patient does not reach a UPDRS III motor score of 30 after a 2 hour waiting time, inhalations tests will start if the UPDRS III motor score is at least 25.

2. OBJECTIVES

The objective of this study is to investigate whether Parkinson's patients in the *off* phase are capable to use a dry powder inhaler correctly, regarding the generation of an appropriate inhalation curve for aerosol generation and transport into the peripheral airways.
For the Twincer™ to be suitable for Parkinson's patients in the *off* period, it has to be known at which inspiratory air flow and inhaled volume the fine particle dose has to be delivered. This may require adjustment of emission time for the fine particle dose and air flow resistance of the inhaler device. The data obtained with the test inhaler (by using 3 resistances) could be used to optimise the Twincer™ DPI for Parkinson's patients. Specific characteristics of the Twincer™ could be adjusted to what Parkinson's patients can do with a DPI, in order to realise a DPI with levodopa that Parkinson's patients can operate during the *off* phase. Such characteristics are for example the resistance, the dose and the emission profile. With this new knowledge we aim to develop a DPI with levodopa for Parkinson's patients.

Primary Objective: to determine if a Parkinson's patient can operate a test inhaler correctly during *off* periods. The parameter that describes whether or not a Parkinson's patient is capable of using the inhaler correctly is the pressure drop (s)he creates over the inhaler upon inhalation (by testing 3 resistances, of which one is comparable with Twincer™ , one higher and lower). From the created pressure drop, various inspiratory parameters can be calculated. For the Twincer™ a pressure drop between 2 kPa and 4 kPa is optimal.

3. STUDY DESIGN

The study is a non-therapeutic observational study in which the applicability of dry powder inhalation in Parkinson's patients will be investigated. The study will be conducted with an instrumented test inhaler. The test inhaler concept is a dummy, without any drug substance or excipient. It is equipped with a pressure drop meter to record the inhalation curve of the patient. A more extensive description of the test inhaler is given in the introduction and in paragraph *5.2 Test inhaler*.

The duration of the study is approximately 12 months (different days) and will be conducted at the Martini Ziekenhuis. Only Parkinson's patients who are under treatment by a neurologist in the Martini Ziekenhuis are able to join. For the individual patient, the test will last maximally 30 minutes (including instruction and motor function examination). The tests will be performed in the Martini Ziekenhuis.

A family member is allowed to be present during the tests. In order to be able to measure during an *off* period, patients will be asked to postpone a dose of levodopa until they become *off*. As soon as the patient starts feeling *off,* explanation of the test procedure will be started. Because becoming *off* is not one fixed point in time but will take a while, a 15 minute waiting period before start of the inhalation test will be taken. During this waiting period, the researcher will explain the test procedure. The patient will receive instructions on how to use the test inhaler. The instructions will be given orally and partly be demonstrated by the instructor, partly be visualised by photographs and recordings of generated flow curves on a computer screen. 15 minutes after the patients started to feel *off*, the motor examination will be performed. We prefer to start the inhalation tests when the UPDRS III motor score is at least 30 (out of 56). However, if the patient does not reach a UPDRS III motor score of 30 after a 2 hour waiting time, inhalations tests will start if the UPDRS III motor score is at least 25. The patient will do a couple of inhalations with three different resistances. After the patient finished the test inhalations, the patient will take his/her levodopa. The patient will leave the hospital only in an *on* period.

All patients will receive the opportunity to do a few test inhalations with the standard test inhaler. During the inhalations the patient can see the flow curves on the computer screen. This gives the opportunity to illustrate and explain how the inhalation manoeuvre can be improved. The data that will be collected per Parkinson's patient consist of a maximum of ten flow curves (for the three resistances together).

4. STUDY POPULATION

4.1 Population (base)

The study will be conducted in 15 Parkinson's patients. The duration of the study is approximately 12 months and will be conducted at the Martini Ziekenhuis. The patients are aged 18 year and older and do have recognizable *off* periods. Informed consent of the patient will be obtained prior to participation. Parkinson's patients with active pulmonary disease are excluded. Patients currently treated with apomorphine or duodopa by pump are also excluded.

4.2 Inclusion criteria (must meet all inclusion criteria)

- Predictable *off* periods.
- Recognizable *off* periods for themselves and others.
- At least 2 years of levodopa use.
- Signed informed consent.
- Diagnosed with Parkinson's disease according to the UK Parkinson's disease society Brain Bank Clinical Diagnostic Criteria.

4.3 Exclusion criteria (excluded if they meet one or more of the following criteria)

- Cognitive dysfunction, which precludes good understanding of instructions and/or informed consent.
- Current treatment with apomorphine or duodopa by pump.
- Active pulmonary disease.

4.4 Sample size calculation

The study is an observational study designed to obtain insight into the ability of Parkinson's patients to use a dry powder inhaler during *off* periods.

We based our sample size calculation on previous studies performed in Parkinson's disease. Canning et al (1997) evaluated exercise capacity, respiratory function and gait in 16 patients with mild to moderate Parkinson's disease. Guedes et al (2012) investigated the MIP and MEP in 26 patients with Parkinson's disease during *on* and *off* periods of levodopa compared to healthy volunteers. Seccombe et al (2011) recruited 19 patients with mild-moderate Parkinson's disease without respiratory disease and assed ventilatory control. Since we only want to obtain insight into the ability of Parkinson's patients to use a dry powder inhaler during *off* periods, 15 patients valid for evaluation is sufficient.

5. METHODS

5.1 Study parameters

5.1.1 Main study parameters

The main study parameter is to determine if a Parkinson's patient can operate a test inhaler correctly during *off* periods, by testing if they can generate a sufficiently large airflow and volume through the test inhaler *theoretically* necessary to disperse a medicinal powder and transport the aerosol into the lower airways during an *off* period.
The parameter that describes whether or not a patient is capable of using the inhaler correctly is the (duration of the) pressure drop (s)he creates over the inhaler upon inhalation. For the Twincer™ a pressure drop between 2 kPa and 4 kPa is optimal. The main study parameter therefore is the capability of patients to create a pressure drop between 2 kPa and 4 kPa by using a test inhaler with the resistance comparable with the Twincer™. At least 11 out of 15 patients should be able to create a pressure drop between 2kPa and 4 kPa, when using the resistance comparable of the Twincer™.

From the pressure drops created with the three resistances, the following parameters (which give more insight in the inhalation profile) will be calculated:

- Peak and mean inspiratory flow rate (PIFR and MIFR).
- Acceleration towards peak inspiratory flow rate (flow increase rate: FIR).
- Inhaled volume.
- Total inhalation time.

The data obtained with the test inhaler (by using the three resistances) could be used to optimise the Twincer™ DPI for Parkinson's patients. Specific characteristics of the Twincer™ could be adjusted to what Parkinson's patients can do with a DPI, in order to realise a DPI with levodopa that Parkinson's patients can operate during the *off* phase. Such characteristics are for example the resistance, the dose and the emission profile. With this new knowledge we aim to develop a DPI with levodopa for Parkinson's patients.

5.2 Test inhaler

The study will be conducted with an instrumented test inhaler with exchangeable air flow resistances. The test inhaler is a dummy, without any drug substance or excipient. The device is provided with a pressure drop meter that records the pressure drop that is generated when the patient inhales through it. The pressure drop meter is linked to a computer equipped with software that calculates from the pressure drop the inspiratory flow rate as function of the inhalation time. Various relevant parameters can be derived from this curve, such as inhaled volume, total inhalation time and mean and peak inspiratory flow rate. In order to adequately break up (disperse) the inhalation powder with the current Twincer™ into particles in a size range suitable for lung deposition, a peak pressure drop within the range of 2 to 4 kPa must be generated. Because the test inhaler can mimic the resistance of the current Twincer™, it can be investigated whether the patients can fulfil this requirement or adjustment of the Twincer™ to the inspiratory capabilities of Parkinson's patients in the off period will be needed. This may depend on the inhaler resistance, and for that reason different resistance values will be tested. The test inhaler is made of polypropylene, a robust and light weight material that does not break, to prevent the formation of inhalable particles (e.g. from dropping, thumping); the exchangeable mouthpiece will be cleaned and disinfected between Parkinson's patients. The design of the test inhaler excludes the inhalation of inhaler parts during the tests. The test inhaler is shown in figure 2 in the introduction. This figure shows that the test inhaler does not resemble the Twincer™. It is a measuring instrument specially designed for inventory studies regarding inspiratory capacities. The test inhaler has been successfully used in another study; (ChildDPI-1) 'Investigation of the applicability of dry powder inhalation in school children' and has therefore been tested for technical and hygienic safety by the medical technique of the UMCG.

5.3 Study procedures

The tests will be performed in the Martini Ziekenhuis. A family member is allowed to be present if desired. The patients will be asked to arrive in the Martini Ziekenhuis around a scheduled time of levodopa administration. In order to be able to measure during an *off* period, patients will be asked to postpone the intake of the scheduled levodopa until they become *off*. As soon as the patient starts feeling *off,* explanation of the test procedure will be started. Because becoming *off* is not one fixed point in time but will take a while, a 15 minute waiting period before start of the inhalation test will be taken. During this waiting period, the researcher will explain the test procedure. The patient will receive instructions

on how to use the test inhaler. The instructions will be given orally and partly be demonstrated by the instructor, partly be visualised by photographs and recordings of generated flow curves on a computer screen. 15 minutes after the patients started to feel *off*, a motor function examination will be performed. We prefer to start the inhalation tests when the UPDRS III motor score is at least 30 (out of 56). However, if the patient does not reach a UPDRS III motor score of 30 after a 2 hour waiting time, inhalations tests will start if the UPDRS III motor score is at least 25. In this study, three resistances will be used for the inhalation tests, (one resistance comparable with the resistance of the Twincer™, one higher and one lower resistance). A maximum of ten flow curves will be collected (for the three resistances together). After they finish the test inhalations, the patients will take their levodopa. They will leave the hospital only when they are in an *on* period.

Test 1: Motor function examination

The Unified Parkinson's Disease Rating Scale (UPDRS) (see appendix 2) is a tool to assess severity of symptoms in Parkinson's disease (Pal et al, 2013). The UPDRS is the primary outcome measure in most clinical trials of Parkinson's disease therapeutics (Tavares et al, 2005). The UPDRS consists of six sections, in which all items (unless otherwise indicated) are rated from zero (normal) to four (severely affected).
The UPDRS consists of six sections, in which all items (unless otherwise indicated) are rated from zero (normal) to four (severely affected).

Since motor symptoms (rigidity, bradykinesia) are major symptoms during an *off* period, the UPDRS III motor examination section will be useful to assess the clinical state of motor function immediately before the inhalation tests will start. The UPDRS motor examination section is a 14-item rating of motor signs. It rates tremor, facial and generalized bradykinesia, and performance on several straightforward tasks (Factor et al, 2002). Each item is rated from zero (normal) to four (severely affected).

In this study, an accredited UPDRS scorer will rate the UPDRS III motor section for each patient, immediately before the inhalation tests will start. This allows the researchers to specify the motor function during inhalation. We prefer to start the inhalation tests when the UPDRS III motor score is at least 30 (severe Parkinson). However, if the patient does not reach a UPDRS III motor score of 30 after a 2 hour waiting time, inhalations tests will start if the UPDRS III motor score is at least 25.

Test 2: Recording of the inhalation curve through the test inhaler with standard mouthpiece and (medium) air flow resistance

The patient will be instructed to first breath out completely (turning the face away from the test inhaler) and then breath in firmly and as long as possible through the test inhaler. Additionally, the patient will be asked to hold the breath for at least 3 to 5 s (if possible). A few inhalations will be practiced before recording of the first curve (and breath hold period) during which the patient can see the flow curve of his/her performance on the computer screen. This gives the opportunity to explain and illustrate how the inhalation manoeuvre can be improved.

When the patient has finished all his/her inhalations (maximum of 10 inhalations; excluding practicing before recording will start), the test inhaler and the mouthpiece that were used will be cleaned and disinfected with ethanol and be allowed to dry prior to using them for the next patient.

5.4 Withdrawal of individual subjects

Subjects can leave the study at any time for any reason if they wish to do so, without any consequences. The investigator can decide to withdraw a subject from the study for urgent medical reasons.

5.5 Replacement of individual subjects after withdrawal

Individual subjects will be replaced after withdrawal, until a number of 15 patients are reached.

5.6 Premature termination of the study

The study will be terminated prematurely if the number of withdrawals is more than 5 of the included Parkinson's patients for whatever reason. This means that when 6 Parkinson's patients are withdrawn from the study during the measurements, the study will be terminated.

6. SAFETY REPORTING

6.1 Section 10 WMO event

In accordance to section 10, subsection 1, of the WMO, the investigator will inform the subjects and the reviewing accredited METC if anything occurs, on the basis of which it appears that the disadvantages of participation may be significantly greater than was foreseen in the research proposal. The study will be suspended pending further review by the accredited METC, except insofar as suspension would jeopardise the subjects' health. The investigator will take care that all subjects are kept informed.

6.2 Adverse and serious adverse events

Despite optimized treatment, many Parkinson's patients still suffer from *off* periods. In the IMPACT registry, which enrolled a large number of patients who were experiencing *off* periods, patients experienced approximately three *off* periods per day. The mean duration of these *off* periods was approximately 50 minutes (Stacy et all, 2006). Most Parkinson's patients are used to have frequent *off* periods.

In order to be able to test the ability to use a dry powder inhaler correctly during an *off* period*,* patients will be asked to postpone the intake of levodopa until they become *off*. Since the half life of levodopa is very short (+/- 90 min) (Nutt, 2008), we expect a patient will become *off* shortly after the planned levodopa dose. As soon as the patient starts feeling *off,* explanation of the test procedure will be started. Because becoming *off* is not one fixed point in time but will take a while, a 15 minute waiting period before start of the inhalation test will be taken. During this waiting period, the researcher will explain the test procedure. 15 minutes after the patients started to feel *off*, the motor function examination will be performed. We prefer to start the inhalation tests when the UPDRS III motor score is at least 30 (out of 56). However, if the patient does not reach a UPDRS III motor score of 30 after a 2 hour waiting time, inhalations tests will start if the UPDRS III motor score is at least 25. After they finish the test inhalations, they will take their levodopa. The total test procedure (explanation, motor function examination, inhalations) will take not more than 30 minutes. Patients will leave the hospital only when they are in an *on* period. Since all patients included are familiar with frequent *off* episodes, no new discomfort compared to other *off* episodes is expected. The timeframe between the morning levodopa dose and the postponed levodopa dose is expected to be shorter than the levodopa free period during the night. The participating patients are therefore used to a levodopa free period and the effect of a levodopa free period on their motor function.

The tests will be performed with a dummy test inhaler without any drug substance or excipient. Therefore, the risks of the test procedure itself are negligible. If a patient experiences unease or discomfort at any time during the test or the exercise inhalations, the test will be stalled immediately. Adverse events are defined as any undesirable experience occurring to a subject during the study, whether or not considered related to the test procedure. All adverse events reported spontaneously by the subject or observed by the investigator or his staff will be recorded. The possibility of the occurrence of serious adverse effects caused by use of the test inhaler is excluded by the use of a dummy test inhaler without drug or excipient.

6.3 Follow-up of adverse events

All adverse events will be followed until they have abated, or until a stable situation has been reached. Depending on the event, follow up may require additional tests or medical procedures as indicated, and/or referral to the general physician or a medical specialist.

7. STATISTICAL ANALYSIS

7.1 Descriptive statistics

Routine descriptive statistics of the subject characteristics (e.g. age, gender, weight, length, UPDRS III score) will be presented. The capability of the Parkinson's patients to generate a sufficiently large pressure drop will be described qualitatively.

8. ETHICAL CONSIDERATIONS

8.1 Regulation statement

This study will be conducted according to the principles of the Declaration of Helsinki (version 9, October 2008, www.wma.net) and in accordance with the Medical Research Involving Human Subjects Act (WMO).

8.2 Recruitment and consent

The study will be conducted in outpatient Parkinson's patients who are treated by a Neurologist of the Martini Ziekenhuis.

A Martini Ziekenhuis neurologist with Parkinson expertise will select possible suitable patients. Patients will be asked to participate by one of the researchers. The patient will receive patient information and an informed consent form from the researchers. The patients will have two weeks to consider their decision. The patient is allowed to contact

an independent physician for advice. The patient, neurologist and researcher must sign the informed consent form if consent is given. A stamped envelope will be enclosed by the patient information and informed consent form that can be used to send the informed consent form(s) to the investigator.

8.3 Benefits and risks assessment, group relatedness

This observational study has no specific benefits for the participating patients. The test inhaler that is used is a dummy, which means it contains neither drug nor excipient. Therefore, the patient will not inhale anything but air during the tests. Furthermore, the design of the test inhaler will exclude the possibility of inhalation of any inhaler part during the tests. The test inhaler is made of polypropylene, a tough and light weight material that does not wear down, to prevent the formation of small, inhalable particles (e.g. due to dropping, thumping or screwing on the resistance disk too tightly). This material is inert to ethanol. Therefore, parts of the inhaler can be cleaned and disinfected with this liquid without degradation of the material. Moreover, we have limited the study procedures to ten inhalations that will be performed in one session of maximally 30 minutes per patient.

In order to be able to test the ability to use a dry powder inhaler correctly during an *off* period, patients will be asked to postpone the intake of levodopa until they become *off*. Since the half life of levodopa is very short (60-90 min), we expect a patient will become *off* shortly after the planned levodopa dose. After they finish the test inhalations, they will take their levodopa. They will leave the hospital only when they are in an *on* period. Since all patients included are familiar with frequent *off* episodes, no new discomfort compared to other *off* episodes is expected. The timeframe between the morning levodopa dose and the postponed levodopa dose is expected to be shorter than the levodopa free period during the night. The patients are used to a levodopa free period and the effect of this levodopa free period on their motor function.

The objectives of the study are group related. Only when performed in Parkinson's patients during an *off* period, specific group related information on the inspiratory capacities of these patients could be obtained.

8.4 Insurance

As explained in the prior paragraph, the study is designed in such a way that the risks of participating are negligible. Therefore, a dispensation from the statutory obligation to provide insurance is requested. This request is enclosed in the research file (G1).

9. ADMINISTRATIVE ASPECTS AND PUBLICATION

9.1 Handling and storage of data and documents

Each patient will be assigned a Patient Allocation Number on registration. The Patient Allocation Number and the patient initials are to be entered on the Case Report From. The investigator will retain all patient information for a period of at least 15 year from study completion. Data will be handled confidentially and if possible anonymously. Where it is necessary to be able to trace data to an individual subject, a subject identification code list will be used to link the data to the subject. The principal investigator will safeguard the key to the code. The handling of personal data will comply with the Dutch Personal Data Protection Act (in Dutch: De Wet Bescherming Persoonsgegevens).

9.2 Amendments

Amendments are changes made to the research after a favourable opinion by the accredited METC has been given. All amendments will be notified to the METC that gave a favourable opinion.

9.3 End of study report

The investigator will notify the accredited METC of the end of the study within a period of 90 days. The end of the study is defined as the last patient's visit.

In case the study is ended prematurely, the investigator will notify the accredited METC, including the reasons for the premature termination.

Within one year after the end of the study, the investigator will submit a final study report with the results of the study, including any publications/abstracts of the study, to the accredited METC.

9.4 Public disclosure and publication policy

Upon completion of the study, appropriate measures will be taken to ensure suitable publicity. The trial will be added to the public registry prior to the start of the study at www.trialregister.nl.

10. STRUCTURED RISK ANALYSIS

10.1 Potential issues of concern

10.1.1 Previous use of the test inhaler

The test inhaler has been successfully used in another study; (ChildDPI-1) 'Investigation of the applicability of dry powder inhalation in school children' and has therefore been tested for technical and hygienic safety by the department of medical technique in the UMCG. Therefore, no problems due to the use of the test inhaler are expected.

10.1.2 Analysis of potential effect.

The test inhaler is a dummy, which means it contains neither drug nor excipient. Therefore, the patient will not inhale anything but air during the tests. Furthermore, the design of the test inhaler will exclude the possibility of inhalation of any inhaler part during the tests. The test inhaler is made of polypropylene, a tough and light weight material that does not wear down, to prevent the formation of small, inhalable particles (e.g. due to dropping, thumping or screwing on the resistance disk too tightly). This material is inert to ethanol. Therefore, parts of the inhaler can be cleaned and disinfected with this liquid without degradation of the material. Moreover, we have limited the study procedures to ten inhalations that will be performed in one session of maximally 30 minutes per patient, including . explanation of the tests and the motor function (UPDRS section III) test.

In order to be able to test the ability to use a dry powder inhaler correctly during an *off* period, patients will be asked to postpone the intake of levodopa until they become *off*. Since the half life of levodopa is very short (60-90 min), we expect a patient will become *off* shortly after the planned levodopa dose. We prefer to start the inhalation tests when the UPDRS III motor score is at least 30 (out of 56). However, if the patient does not reach a UPDRS III motor score of 30 after a 2 hour waiting time, inhalations tests will start if the UPDRS III motor score is at least 25. After finishing the test inhalations, which will be performed in maximally 15 minutes, the patient will take his/her levodopa. Since all patients included are familiar with frequent *off* episodes, no new discomfort compared to other *off* episodes is expected. The timeframe between the morning levodopa dose and the postponed levodopa dose is expected to be shorter than the levodopa free period during the

night. The patients are used to a levodopa free period and the effect of this levodopa free period on their motor function.

10.1.3 Study population

The study population consists of Parkinson's patients familiar with *off* periods.

10.1.4 Can effects be managed?

Testing will take place in the hospital. In the hospital, a neurologist will be available in case of emergencies.

10.2 Synthesis

Since participating patients are used to frequent *off* episodes, a levodopa free period and the effect of this levodopa free period on their motor function and since they will not inhale anything but air during the test, the risk of participation are negligible.
In order to reduce eventual risks, patient with significant lung disease are excluded.

11. REFERENCES

BARTUS, R.T. et al, 2004. A pulmonary formulation of L-Dopa enhances effectiveness in a Rat Model of Parkinson's disease. *The journal of pharmacology and experimental therapeutics*. Vol 310, no 2.

CANNING, C.G. et al,1997. Parkinson's disease: An investigation of exercise capacity, respiratory function, and gait. *Arch Phys Med Rehabil Vol 78,* February 1997.

CHEN, J.J. et al, 2005. A review of intermittend subcutaneous apomorphine injections for the rescue management of motor fluctuations associated with advanced Parkinson's disease. *Clinical Therapeutics.* Vol 26, no 11.

CONTIN, M. et al, 1999. Concentration-effect relationship of levodopa-benserazide dispersible formulation versus standard form in the treatment of complicated motor response fluctuations in Parkinson's disease. *Clinical neuropharmacology*, Vol. 22, No 6, pp 351-355.

DE BOER, A.H. et al, 2006. Design and in vitro performance testing of multiple air classifier technology in a new disposable inhaler concept (Twincer) for high powder doses. *Eur. J. Pharm. Sci.,* 28**,** 171-8.

DOI, H. et al, 2012. Plasma levodopa peak delay and impaired gastric emptying in Parkinson's disease. *Journal of the neurological sciences* 319 (2012) 86-88.

FACTOR, S.A. et al. Parkinson's disease: diagnosis and clinical management. *New York: Demos Medical Publishing; 2002. ISBN-10: 1-888799-50-1.* Chapter 14: Quantitative measures and Rating Scales, Stephen T. Gancher.

GOOLE, J, et al, 2009. Levodopa delivery systems for the treatment of Parkinson's disease: An overview. *International Journal of Pharmaceutics* 380 (2009), 1-15.

GUEDES et al, 2012. Respiratory changes in Parkinson's disease may be unrelated to dopaminergic dysfynction. *Arq Neuropsiquiatr* 2012;70(11):847-851.

JANKOVIC et al, 2007. Medical management of levodopa-associated motor complications in patients with Parkinson's disease. *CNS Drugs* 2007; 21 (8): 677-692.

NUTT JG. Pharmacokinetics and pharmacodynamics of Levodopa. *Movement disorders*, Vol, 23. Suppl. 3, 2008, pp. S580-S584.

NYHOLM, D, 2006. Pharmacokinetic optimisation in the treatment of Parkinson's disease, an update. *Clinic Pharmacokinet* ; 45 (2): 109-136.

PAL, G et al, 2013. Assessing bradykinesia in Parkinsonian disorders. *Frontiers in neurology.* June 2013, volume 4, article 54.

SECCOMBE, LM. et al, 2011. Abnormal ventilatory control in Parkinson's disease-Further evidence for non-motor dysfunction. *Respiratory Physiology & Neurobiology* 179 (2011) 300-304.

STACY, M. et al, 2008. Apomorphine for the acute treatment of "off" episodes in Parkinson's disease. *Parkinsonism and related disorders* 14 (2008) 85 - 92.

TAVARES, AL. et al, 2005. Quantitative measurements of alternating finger tapping in Parkinson's disease correlate with UPDRS motor disability and reveal the improvement in fine motor control from medication and deep brain stimulation. *Movement disorders*, Vol. 20, No. 10, 2005, pp. 1286-1298.

Ul HAQ, I. et al, 2007. Apomorphine therapy in Parkinson's disease: a review. *Expert Opin. Pharmacother.* (2007) 8(16):2799-2809.

APPENDIX 1

The relationships between air flow resistance, flow rate, aerosol generation and lung deposition

Dry powder inhalers can be considered as resistances to air flow. The driving force for an air flow (Φ) through an inhaler is a pressure drop (dP), as obtained during inhalation when an under pressure in the lungs is created relative to the (ambient) air pressure by expansion of the lung volume. Because the air flow resistance of a powder inhaler is of the orifice type, there exists a linear relationship between the square root of the pressure drop across the inhaler and the flow rate: $\sqrt{dP} = \Phi.R$, in which R represents the air flow resistance. From the equation it can be concluded that for a given pressure drop dP, the flow rate (Φ) increases when the air flow resistance (R) decreases. Due to a high flow rate into the lungs, a large difference between lung pressure and ambient pressure (and thus, a high pressure drop across the inhaler) cannot be maintained.

For passive dry powder inhalers, the energy for aerosol generation is derived from the inhaled air flow through the inhaler. However, it is not the flow rate in absolute sense that determines the fine particle dose from a dry powder inhaler, but the flow rate in relation to the design of the inhaler. Effective inhalers convert the kinetic energy of the air flow into powder dispersion forces and they need high local air velocities within the inhaler for that rather than a high air flow rate through the inhaler. High flow rates through an inhaler are highly unwanted because they increase mouth and throat deposition of the aerosol and shift the deposition in the lungs to larger airways. The way to achieve high local velocities without having a high total air flow rate is to design narrow tortuous channels, turbulent zones or whirl chambers, which all contribute to a higher air flow resistance of the inhaler. Therefore, the prerequisite for operating a dry powder inhaler correctly is rather to achieve sufficient pressure drop across the inhaler than to achieve sufficient flow rate through the device. Currently marketed dry powder inhalers all perform well at a pressure drop between 2 and 4 kPa. They do have different air flow resistances, meaning that the aerosol can be delivered at flow rates varying between 45 and 110 L/min at the same pressure drop of 4 kPa across the device. This will result in different deposition patterns; deepest lung penetration and highest peripheral deposition fractions will be obtained for the inhalers with the highest air flow resistance. Therefore, a high air flow resistance is desirable, not only from deposition point of view, but as explained, also because they facilitate to generate a high pressure drop which also can be maintained over a longer period.

APPENDIX 2 UPDRS (section III) motor examination

III. MOTOR EXAMINATION

18. Speech
0 = Normal.
1 = Slight loss of expression, diction and/or volume.
2 = Monotone, slurred but understandable; moderately impaired.
3 = Marked impairment, difficult to understand.
4 = Unintelligible.

19. Facial Expression
0 = Normal.
1 = Minimal hypomimia, could be normal "Poker Face".
2 = Slight but definitely abnormal diminution of facial expression.
3 = Moderate hypomimia; lips parted some of the time.
4 = Masked or fixed facies with severe or complete loss of facial expression;
lips parted 1/4 inch or more.

20. Tremor at rest (head, upper and lower extremities)
0 = Absent.
1 = Slight and infrequently present.
2 = Mild in amplitude and persistent. Or moderate in amplitude, but only intermittently present.
3 = Moderate in amplitude and present most of the time.
4 = Marked in amplitude and present most of the time.

21. Action or Postural Tremor of hands
0 = Absent.
1 = Slight; present with action.
2 = Moderate in amplitude, present with action.
3 = Moderate in amplitude with posture holding as well as action.
4 = Marked in amplitude; interferes with feeding.

22. Rigidity (Judged on passive movement of major joints with patient relaxed in sitting position. Cogwheeling to be ignored.)
0 = Absent.
1 = Slight or detectable only when activated by mirror or other movements.
2 = Mild to moderate.
3 = Marked, but full range of motion easily achieved.
4 = Severe, range of motion achieved with difficulty.

23. Finger Taps (Patient taps thumb with index finger in rapid succession.)
0 = Normal.
1 = Mild slowing and/or reduction in amplitude.
2 = Moderately impaired. Definite and early fatiguing. May have occasional arrests in movement.
3 = Severely impaired. Frequent hesitation in initiating movements or arrests in ongoing movement.
4 = Can barely perform the task.

24. Hand Movements (Patient opens and closes hands in rapid succesion.)
0 = Normal.
1 = Mild slowing and/or reduction in amplitude.
2 = Moderately impaired. Definite and early fatiguing. May have occasional arrests in movement.
3 = Severely impaired. Frequent hesitation in initiating movements or arrests in ongoing movement.
4 = Can barely perform the task.

25. Rapid Alternating Movements of Hands (Pronation-supination movements of hands, vertically and horizontally, with as large an amplitude as possible, both hands simultaneously.)
0 = Normal.
1 = Mild slowing and/or reduction in amplitude.
2 = Moderately impaired. Definite and early fatiguing. May have occasional arrests in movement.
3 = Severely impaired. Frequent hesitation in initiating movements or arrests in ongoing movement.
4 = Can barely perform the task.

26. Leg Agility (Patient taps heel on the ground in rapid succession picking up entire leg. Amplitude should be at least 3 inches.)
0 = Normal.
1 = Mild slowing and/or reduction in amplitude.
2 = Moderately impaired. Definite and early fatiguing. May have occasional arrests in movement.
3 = Severely impaired. Frequent hesitation in initiating movements or arrests in ongoing movement.
4 = Can barely perform the task.

27. Arising from Chair
(Patient attempts to rise from a straightbacked chair, with arms folded across chest.)
0 = Normal.
1 = Slow; or may need more than one attempt.
2 = Pushes self up from arms of seat.
3 = Tends to fall back and may have to try more than one time, but can get up without help.
4 = Unable to arise without help.

28. Posture
0 = Normal erect.
1 = Not quite erect, slightly stooped posture; could be normal for older person.
2 = Moderately stooped posture, definitely abnormal; can be slightly leaning to one side.
3 = Severely stooped posture with kyphosis; can be moderately leaning to one side.
4 = Marked flexion with extreme abnormality of posture.

29. Gait
0 = Normal.
1 = Walks slowly, may shuffle with short steps, but no festination (hastening steps) or propulsion.
2 = Walks with difficulty, but requires little or no assistance; may have some festination, short steps, or propulsion.
3 = Severe disturbance of gait, requiring assistance.
4 = Cannot walk at all, even with assistance.

30. Postural Stability (Response to sudden, strong posterior displacement produced by pull on shoulders while patient erect with eyes open and feet slightly apart. Patient is prepared.)
0 = Normal.
1 = Retropulsion, but recovers unaided.
2 = Absence of postural response; would fall if not caught by examiner.
3 = Very unstable, tends to lose balance spontaneously.
4 = Unable to stand without assistance.

31. Body Bradykinesia and Hypokinesia (Combining slowness, hesitancy, decreased armswing, small amplitude, and poverty of movement in general.)
0 = None.
1 = Minimal slowness, giving movement a deliberate character; could be normal for some persons. Possibly reduced amplitude.
2 = Mild degree of slowness and poverty of movement which is definitely abnormal. Alternatively, some reduced amplitude.
3 = Moderate slowness, poverty or small amplitude of movement.
4 = Marked slowness, poverty or small amplitude of movement.
